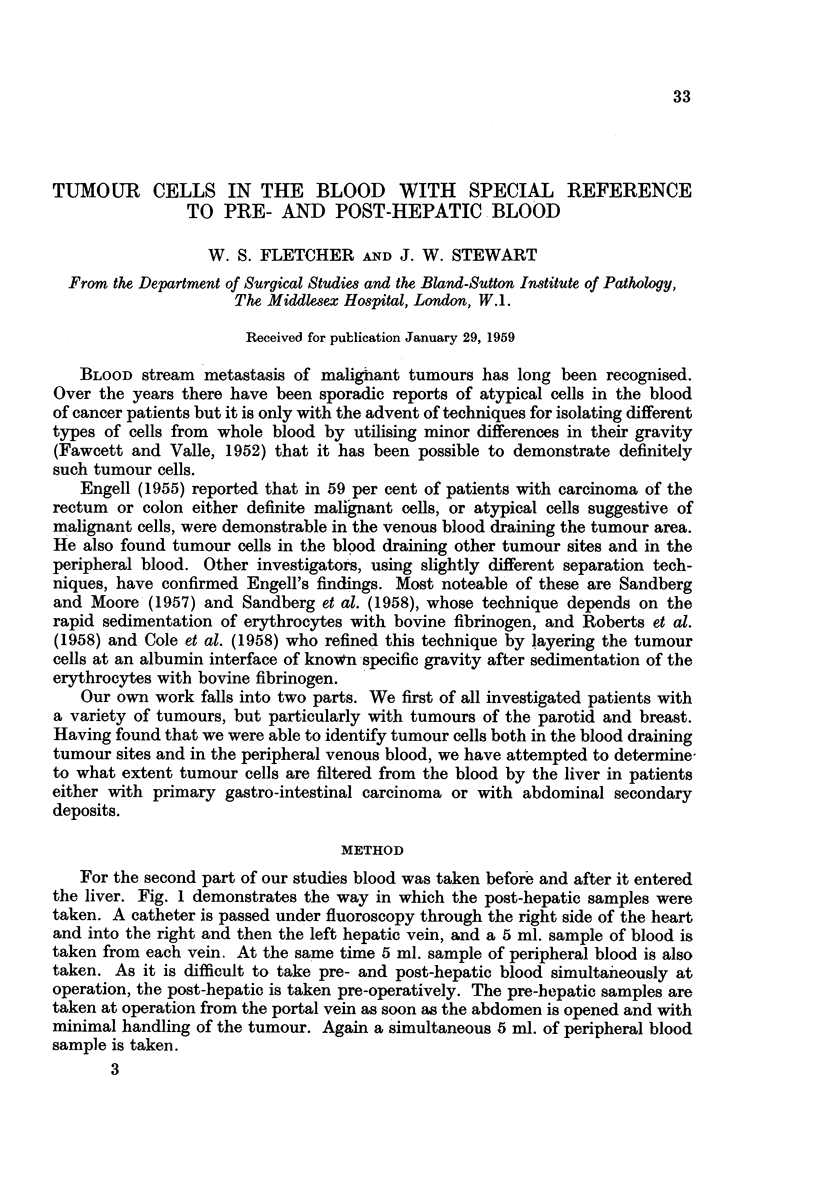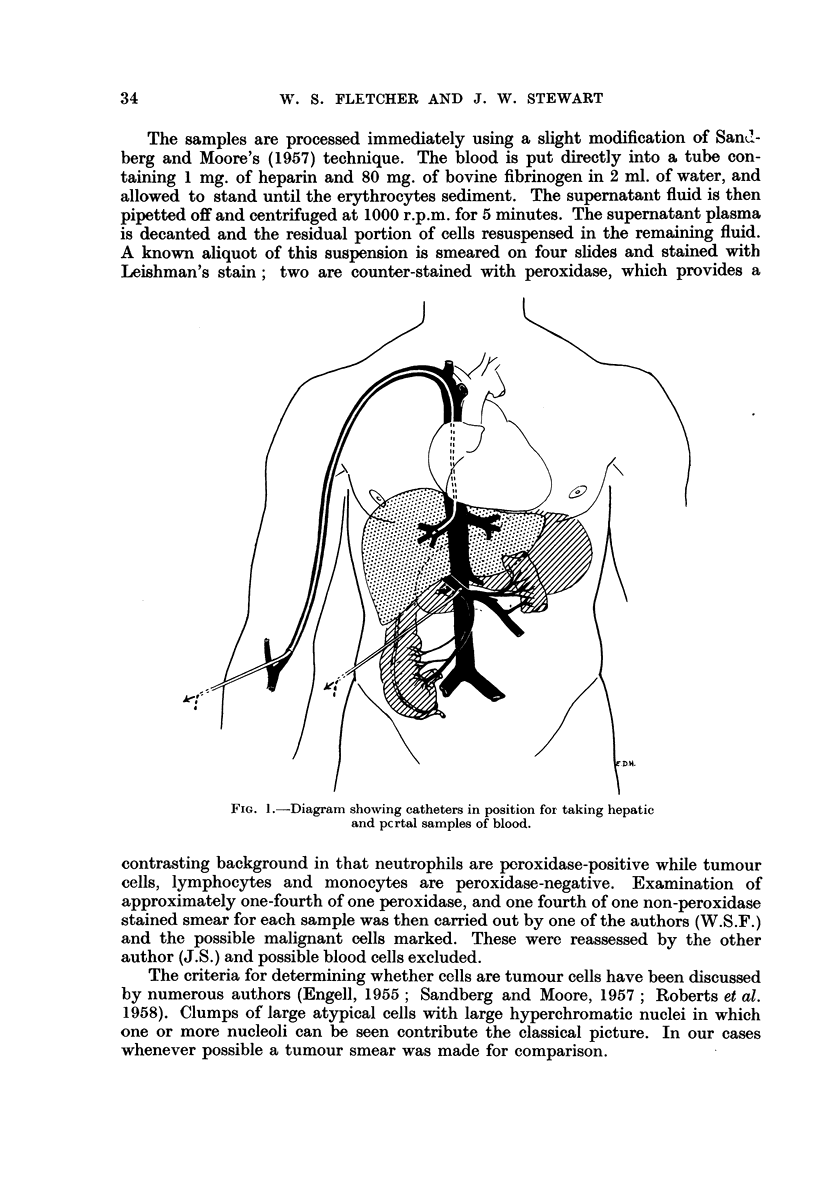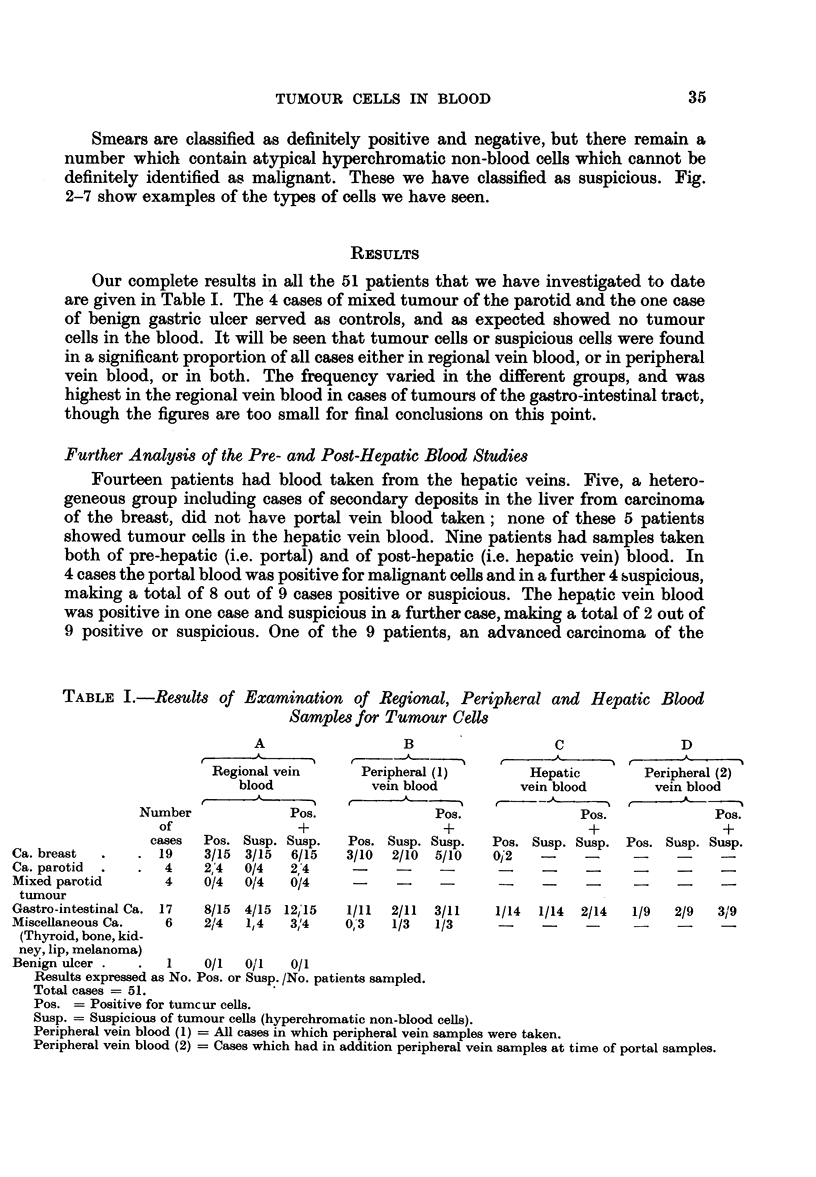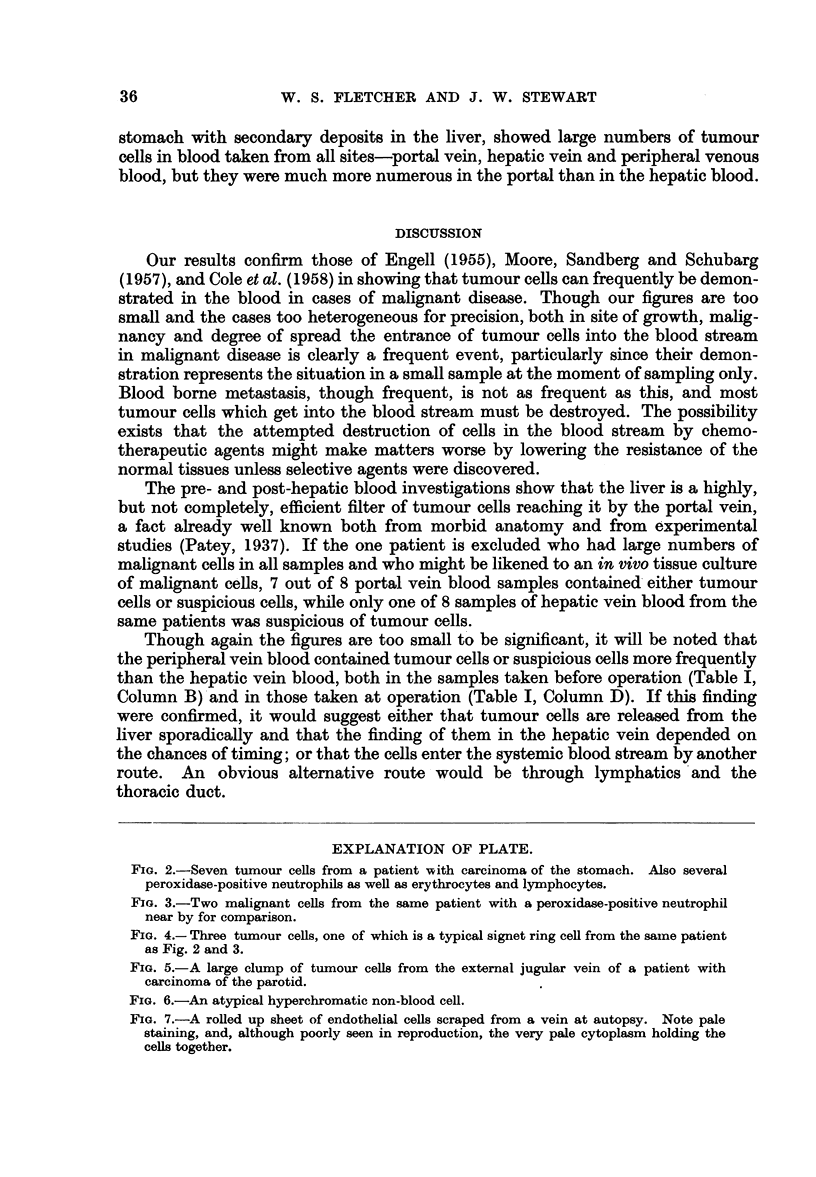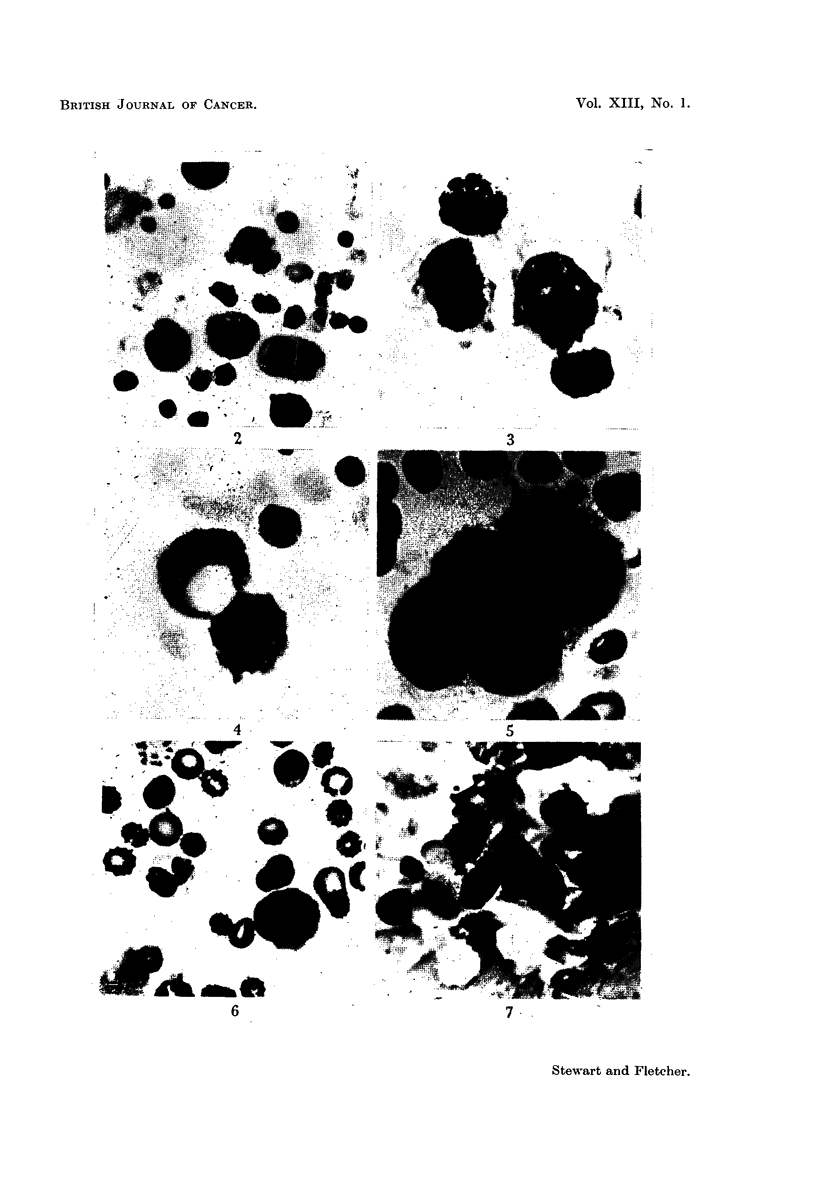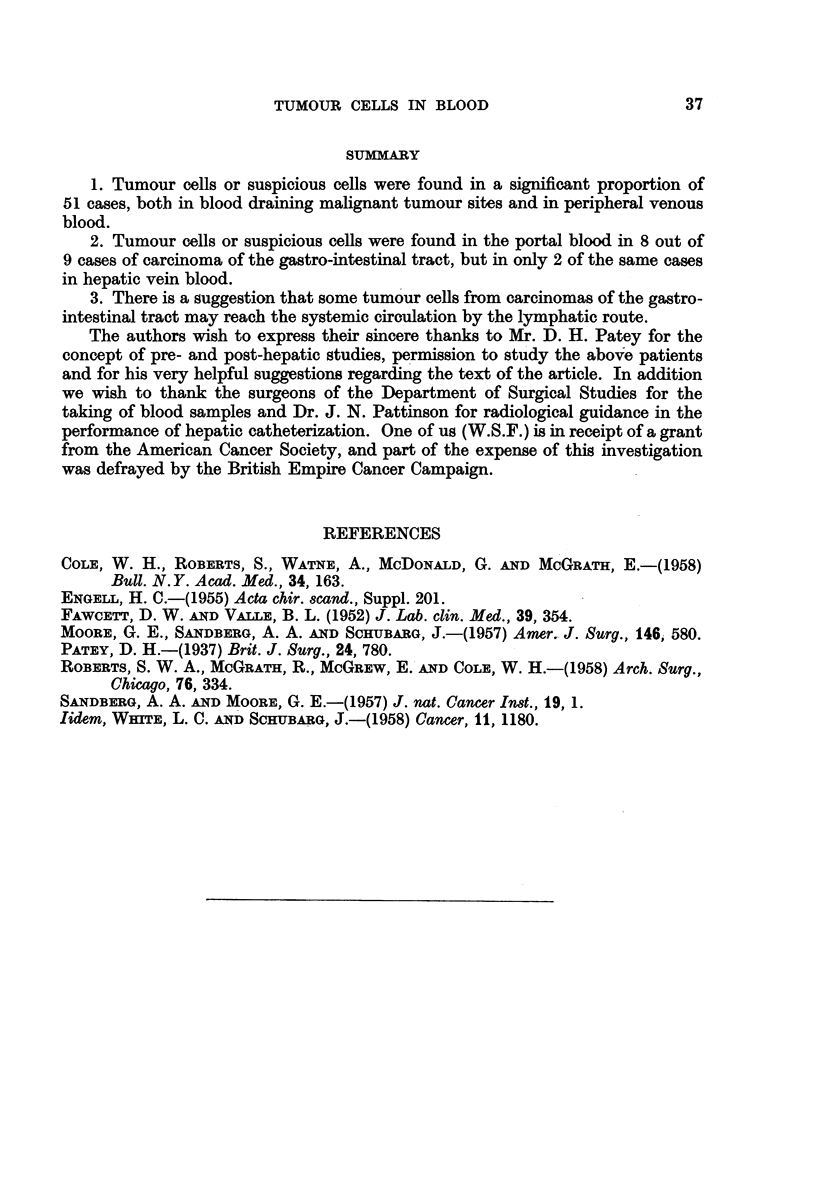# Tumour Cells in the Blood with Special Reference to Pre- and Post-Hepatic Blood

**DOI:** 10.1038/bjc.1959.5

**Published:** 1959-03

**Authors:** W. S. Fletcher, J. W. Stewart

## Abstract

**Images:**


### TUMOUR CELLS IN THE BLOOD WITH SPECIAL REFERENCE TO PRE- AND POST-HEPATIC BLOOD

W. S. FLETCHER AND J. W. STEWART

*From the Department of Surgical Studies and the Bland-Sutton Institute of Pathology, The Middlesex Hospital, London, W.1.*

Received for publication January 29, 1959

BLOOD stream metastasis of malignant tumours has long been recognised. Over the years there have been sporadic reports of atypical cells in the blood of cancer patients but it is only with the advent of techniques for isolating different types of cells from whole blood by utilising minor differences in their gravity (Fawcett and Valle, 1952) that it has been possible to demonstrate definitely such tumour cells.

Engell (1955) reported that in 59 per cent of patients with carcinoma of the rectum or colon either definite malignant cells, or atypical cells suggestive of malignant cells, were demonstrable in the venous blood draining the tumour area. He also found tumour cells in the blood draining other tumour sites and in the peripheral blood. Other investigators, using slightly different separation techniques, have confirmed Engell's findings. Most noteable of these are Sandberg and Moore (1957) and Sandberg *et al.* (1958), whose technique depends on the rapid sedimentation of erythrocytes with bovine fibrinogen, and Roberts *et al.* (1958) and Cole *et al.* (1958) who refined this technique by layering the tumour cells at an albumin interface of known specific gravity after sedimentation of the erythrocytes with bovine fibrinogen.

Our own work falls into two parts. We first of all investigated patients with a variety of tumours, but particularly with tumours of the parotid and breast. Having found that we were able to identify tumour cells both in the blood draining tumour sites and in the peripheral venous blood, we have attempted to determine to what extent tumour cells are filtered from the blood by the liver in patients either with primary gastro-intestinal carcinoma or with abdominal secondary deposits.

### METHOD

For the second part of our studies blood was taken before and after it entered the liver. Fig. 1 demonstrates the way in which the post-hepatic samples were taken. A catheter is passed under fluoroscopy through the right side of the heart and into the right and then the left hepatic vein, and a 5 ml. sample of blood is taken from each vein. At the same time 5 ml. sample of peripheral blood is also taken. As it is difficult to take pre- and post-hepatic blood simultaneously at operation, the post-hepatic is taken pre-operatively. The pre-hepatic samples are taken at operation from the portal vein as soon as the abdomen is opened and with minimal handling of the tumour. Again a simultaneous 5 ml. of peripheral blood sample is taken.

The samples are processed immediately using a slight modification of Sandberg and Moore's (1957) technique. The blood is put directly into a tube containing 1 mg. of heparin and 80 mg. of bovine fibrinogen in 2 ml. of water, and allowed to stand until the erythrocytes sediment. The supernatant fluid is then pipetted off and centrifuged at 1000 r.p.m. for 5 minutes. The supernatant plasma is decanted and the residual portion of cells resuspensed in the remaining fluid. A known aliquot of this suspension is smeared on four slides and stained with Leishman's stain ; two are counter-stained with peroxidase, which provides a

FIG. 1.—Diagram showing catheters in position for taking hepatic and portal samples of blood.

contrasting background in that neutrophils are peroxidase-positive while tumour cells, lymphocytes and monocytes are peroxidase-negative. Examination of approximately one-fourth of one peroxidase, and one fourth of one non-peroxidase stained smear for each sample was then carried out by one of the authors (W.S.F.) and the possible malignant cells marked. These were reassessed by the other author (J.S.) and possible blood cells excluded.

The criteria for determining whether cells are tumour cells have been discussed by numerous authors (Engell, 1955 ; Sandberg and Moore, 1957 ; Roberts *et al.* 1958). Clumps of large atypical cells with large hyperchromatic nuclei in which one or more nucleoli can be seen contribute the classical picture. In our cases whenever possible a tumour smear was made for comparison.

Smears are classified as definitely positive and negative, but there remain a number which contain atypical hyperchromatic non-blood cells which cannot be definitely identified as malignant. These we have classified as suspicious. Fig. 2–7 show examples of the types of cells we have seen.

### Results

Our complete results in all the 51 patients that we have investigated to date are given in Table I. The 4 cases of mixed tumour of the parotid and the one case of benign gastric ulcer served as controls, and as expected showed no tumour cells in the blood. It will be seen that tumour cells or suspicious cells were found in a significant proportion of all cases either in regional vein blood, or in peripheral vein blood, or in both. The frequency varied in the different groups, and was highest in the regional vein blood in cases of tumours of the gastro-intestinal tract, though the figures are too small for final conclusions on this point.

### *Further Analysis of the Pre- and Post-Hepatic Blood Studies*

Fourteen patients had blood taken from the hepatic veins. Five, a heterogeneous group including cases of secondary deposits in the liver from carcinoma of the breast, did not have portal vein blood taken ; none of these 5 patients showed tumour cells in the hepatic vein blood. Nine patients had samples taken both of pre-hepatic (i.e. portal) and of post-hepatic (i.e. hepatic vein) blood. In 4 cases the portal blood was positive for malignant cells and in a further 4 suspicious, making a total of 8 out of 9 cases positive or suspicious. The hepatic vein blood was positive in one case and suspicious in a further case, making a total of 2 out of 9 positive or suspicious. One of the 9 patients, an advanced carcinoma of the

TABLE I.—*Results of Examination of Regional, Peripheral and Hepatic Blood Samples for Tumour Cells*

| | | A | | | B | | | C | | | D | | |
|---|---|---|---|---|---|---|---|---|---|---|---|---|---|
| | | Regional vein blood | | | Peripheral (1) vein blood | | | Hepatic vein blood | | | Peripheral (2) vein blood | | |
| | Number of cases | Pos. | Susp. | Pos. + Susp. | Pos. | Susp. | Pos. + Susp. | Pos. | Susp. | Pos. + Susp. | Pos. | Susp. | Pos. + Susp. |
| Ca. breast . . | 19 | 3/15 | 3/15 | 6/15 | 3/10 | 2/10 | 5/10 | 0/2 | — | — | — | — | — |
| Ca. parotid . . | 4 | 2,4 | 0/4 | 2,4 | — | — | — | — | — | — | — | — | — |
| Mixed parotid tumour | 4 | 0/4 | 0/4 | 0/4 | — | — | — | — | — | — | — | — | — |
| Gastro-intestinal Ca. | 17 | 8/15 | 4/15 | 12,15 | 1/11 | 2/11 | 3/11 | 1/14 | 1/14 | 2/14 | 1/9 | 2/9 | 3/9 |
| Miscellaneous Ca. (Thyroid, bone, kidney, lip, melanoma) | 6 | 2/4 | 1,4 | 3,4 | 0,3 | 1/3 | 1/3 | — | — | — | — | — | — |
| Benign ulcer . . | 1 | 0/1 | 0/1 | 0/1 | | | | | | | | | |

Results expressed as No. Pos. or Susp. /No. patients sampled.
Total cases = 51.
Pos. = Positive for tumcur cells.
Susp. = Suspicious of tumour cells (hyperchromatic non-blood cells).
Peripheral vein blood (1) = All cases in which peripheral vein samples were taken.
Peripheral vein blood (2) = Cases which had in addition peripheral vein samples at time of portal samples.

stomach with secondary deposits in the liver, showed large numbers of tumour cells in blood taken from all sites—portal vein, hepatic vein and peripheral venous blood, but they were much more numerous in the portal than in the hepatic blood.

### DISCUSSION

Our results confirm those of Engell (1955), Moore, Sandberg and Schubarg (1957), and Cole *et al.* (1958) in showing that tumour cells can frequently be demonstrated in the blood in cases of malignant disease. Though our figures are too small and the cases too heterogeneous for precision, both in site of growth, malignancy and degree of spread the entrance of tumour cells into the blood stream in malignant disease is clearly a frequent event, particularly since their demonstration represents the situation in a small sample at the moment of sampling only. Blood borne metastasis, though frequent, is not as frequent as this, and most tumour cells which get into the blood stream must be destroyed. The possibility exists that the attempted destruction of cells in the blood stream by chemotherapeutic agents might make matters worse by lowering the resistance of the normal tissues unless selective agents were discovered.

The pre- and post-hepatic blood investigations show that the liver is a highly, but not completely, efficient filter of tumour cells reaching it by the portal vein, a fact already well known both from morbid anatomy and from experimental studies (Patey, 1937). If the one patient is excluded who had large numbers of malignant cells in all samples and who might be likened to an *in vivo* tissue culture of malignant cells, 7 out of 8 portal vein blood samples contained either tumour cells or suspicious cells, while only one of 8 samples of hepatic vein blood from the same patients was suspicious of tumour cells.

Though again the figures are too small to be significant, it will be noted that the peripheral vein blood contained tumour cells or suspicious cells more frequently than the hepatic vein blood, both in the samples taken before operation (Table I, Column B) and in those taken at operation (Table I, Column D). If this finding were confirmed, it would suggest either that tumour cells are released from the liver sporadically and that the finding of them in the hepatic vein depended on the chances of timing; or that the cells enter the systemic blood stream by another route. An obvious alternative route would be through lymphatics and the thoracic duct.

---

### EXPLANATION OF PLATE.

FIG. 2.—Seven tumour cells from a patient with carcinoma of the stomach. Also several peroxidase-positive neutrophils as well as erythrocytes and lymphocytes.

FIG. 3.—Two malignant cells from the same patient with a peroxidase-positive neutrophil near by for comparison.

FIG. 4.— Three tumour cells, one of which is a typical signet ring cell from the same patient as Fig. 2 and 3.

FIG. 5.—A large clump of tumour cells from the external jugular vein of a patient with carcinoma of the parotid.

FIG. 6.—An atypical hyperchromatic non-blood cell.

FIG. 7.—A rolled up sheet of endothelial cells scraped from a vein at autopsy. Note pale staining, and, although poorly seen in reproduction, the very pale cytoplasm holding the cells together.

2

3

4

5

6

7

SUMMARY

1. Tumour cells or suspicious cells were found in a significant proportion of 51 cases, both in blood draining malignant tumour sites and in peripheral venous blood.

2. Tumour cells or suspicious cells were found in the portal blood in 8 out of 9 cases of carcinoma of the gastro-intestinal tract, but in only 2 of the same cases in hepatic vein blood.

3. There is a suggestion that some tumour cells from carcinomas of the gastro-intestinal tract may reach the systemic circulation by the lymphatic route.

The authors wish to express their sincere thanks to Mr. D. H. Patey for the concept of pre- and post-hepatic studies, permission to study the above patients and for his very helpful suggestions regarding the text of the article. In addition we wish to thank the surgeons of the Department of Surgical Studies for the taking of blood samples and Dr. J. N. Pattinson for radiological guidance in the performance of hepatic catheterization. One of us (W.S.F.) is in receipt of a grant from the American Cancer Society, and part of the expense of this investigation was defrayed by the British Empire Cancer Campaign.

### REFERENCES

Cole, W. H., Roberts, S., Watne, A., McDonald, G. and McGrath, E.—(1958) *Bull. N.Y. Acad. Med.*, **34**, 163.

Engell, H. C.—(1955) *Acta chir. scand.*, Suppl. 201.

Fawcett, D. W. and Valle, B. L. (1952) *J. Lab. clin. Med.*, **39**, 354.

Moore, G. E., Sandberg, A. A. and Schubarg, J.—(1957) *Amer. J. Surg.*, **146**, 580.

Patey, D. H.—(1937) *Brit. J. Surg.*, **24**, 780.

Roberts, S. W. A., McGrath, R., McGrew, E. and Cole, W. H.—(1958) *Arch. Surg., Chicago*, **76**, 334.

Sandberg, A. A. and Moore, G. E.—(1957) *J. nat. Cancer Inst.*, **19**, 1.

*Iidem*, White, L. C. and Schubarg, J.—(1958) *Cancer*, **11**, 1180.